# Surface plasmon resonance enhancement of photoluminescence intensity and bioimaging application of gold nanorod@CdSe/ZnS quantum dots

**DOI:** 10.3762/bjnano.10.3

**Published:** 2019-01-03

**Authors:** Siyi Hu, Yu Ren, Yue Wang, Jinhua Li, Junle Qu, Liwei Liu, Hanbin Ma, Yuguo Tang

**Affiliations:** 1 CAS Key Laboratory of Bio-Medical Diagnostics, Suzhou Institute of Biomedical Engineering and Technology, Chinese Academy of Sciences, Suzhou 215163, P. R. China; 2 School of Science, Changchun University of Science and Technology, Changchun 130022, P. R. China; 3 College of Optoelectronic Engineering, Shenzhen University, Shenzhen 518060, P. R. China

**Keywords:** bioimaging, gold nanorods, photoluminescence enhancement, quantum dots

## Abstract

Biological applications of core/shell near-infrared quantum dots (QDs) have attracted broad interest due to their unique optical and chemical properties. Additionally, the use of multifunctional nanomaterials with near-infrared QDs and plasmonic functional nanoparticles are promising for applications in electronics, bioimaging, energy, and environmental-related studies. In this work, we experimentally demonstrate how to construct a multifunctional nanoparticle comprised of CdSe/ZnS QDs and gold nanorods (GNRs) where the GNRs were applied to enhance the photoluminescence (PL) of the CdSe/ZnS QDs. In particular, we have obtained the scattering PL spectrum of a single CdSe/ZnS QD and GNR@CdSe/ZnS nanoparticle and comparison results show that the CdSe/ZnS QDs have an apparent PL enhancement of four-times after binding with GNRs. In addition, in vitro experimental results show that the biostability of the GNR@CdSe/ZnS nanoparticles can be improved by using folic acid. A bioimaging study has also been performed where GNR@CdSe/ZnS nanoparticles were used as an optical process for MCF-7 breast cancer cells.

## Introduction

In the past decades, quantum dots (QDs) have proven to be increasingly useful for their unique features [1–5]. The light emission from QDs can be easily tuned from the visible to the near-infrared (NIR) spectrum. QDs have emerged as the next generation of luminescence materials, and they have been widely used as nanoprobes for bioimaging and biosensing. As semiconductor nanocrystals, their large surface-to-volume ratio is advantageous and they show excellent quantum confinement for charge carrier and lattice mismatch. In such cases, the energy range of the QDs depends on the relative conduction and valence band offsets for the two materials, and this characteristic can enable many chemical materials or biomolecules to conjugate with them [6–12]. Research conducted to date shows that the fluorescence intensity of QDs changes when other chemical materials or ions are added. The CdSe/ZnS heterostructures of QDs are of interest due to their high quantum efficiency [13–15].

Furthermore, the heterostructure formed with metals and semiconductors, i.e., plasmonic, composite QD nanostructures, provides another efficient way to tune the unique optical properties. In the past decades, much attention has been given to the development of metal-enhanced optical properties. Some researchers have noted that certain metal materials play a role in the enhancement of fluorescence in QDs, especially gold nanorods (GNRs) and Cu or Ca^+^ ion binding of QDs [16–19].

GNRs possess two plasmonic resonance bands – a longitudinal band and a transverse band. These bands correspond to the electron oscillations along the long and short axes of the particle. Their surfaces can be easily modified with other chemicals due to the large area-to-volume ratio of GNRs. Also, there is a positive charge coating on the GNR surface, so it is easily conjugated with QDs due to the negative charge of the QD surface. The excellent stability and biocompatibility of GNRs has been reported by several researchers, and they are being investigated as a probe for photothermal therapy in nanomedicine. The presence of longitudinal surface plasmon resonance (LSPR) provides GNRs with richer optical properties, which lead to local field, Raman, and fluorescence enhancement. When QDs bind with GNRs, the fluorescence intensity of the QDs is enhanced by the near-fired plasmonic resonance from the GNR surface and their photoluminescence (PL) emission increases [20–23].

Richard A. Vaia and co-authors reported a five times higher fluorescence enhancement by organizing QDs on GNRs, and Anjali Kshirsagar et al. reported the electronic structure of free-standing and gold-attached passivated CdSe nanorods [24–25]. These studies covered the synthesis of GNR@CdSe/ZnS nanoparticles using different methods; however, most of these synthesis methods are complicated, and it is difficult to reproduce their preparation and desired applications. It is desirable to find a simple method to synthesize GNR@CdSe/ZnS nanoparticles in aqueous-phase which exhibit high PL emission and optimize the bioconjugated surfaces of these nanoparticles for biological application and therapy of human diseases [26–27].

In this work, we demonstrated a novel GNR@CdSe/ZnS multimodal nanostructure in aqueous phase. We chose CdSe/ZnS QDs as a PL contributor due to its high degree of brightness, excellent photostability, and good spectral overlap with GNRs. We then used the GNRs to enhance the PL intensity of the CdSe/ZnS QDs. The PL from GNR@CdSe/ZnS nanoparticles is approximately four times more than that from CdSe/ZnS QDs. Finite difference time domain (FDTD) simulations were also conducted to understand the plasmon coupling effect on PL enhancement. Additionally, we investigated the PL signal from a single particle, which indicated a stronger PL intensity compared to that of CdSe/ZnS QDs alone. We also prepared the GNR@CdSe/ZnS modified with folic acid (FA) in cell culture for biological applications. These studies indicated that these multifunctional nanoparticles were of low toxicity and had bright luminescence, which make them suitable for biosensor and optical detection studies.

## Results and Discussion

**Figure 1:**
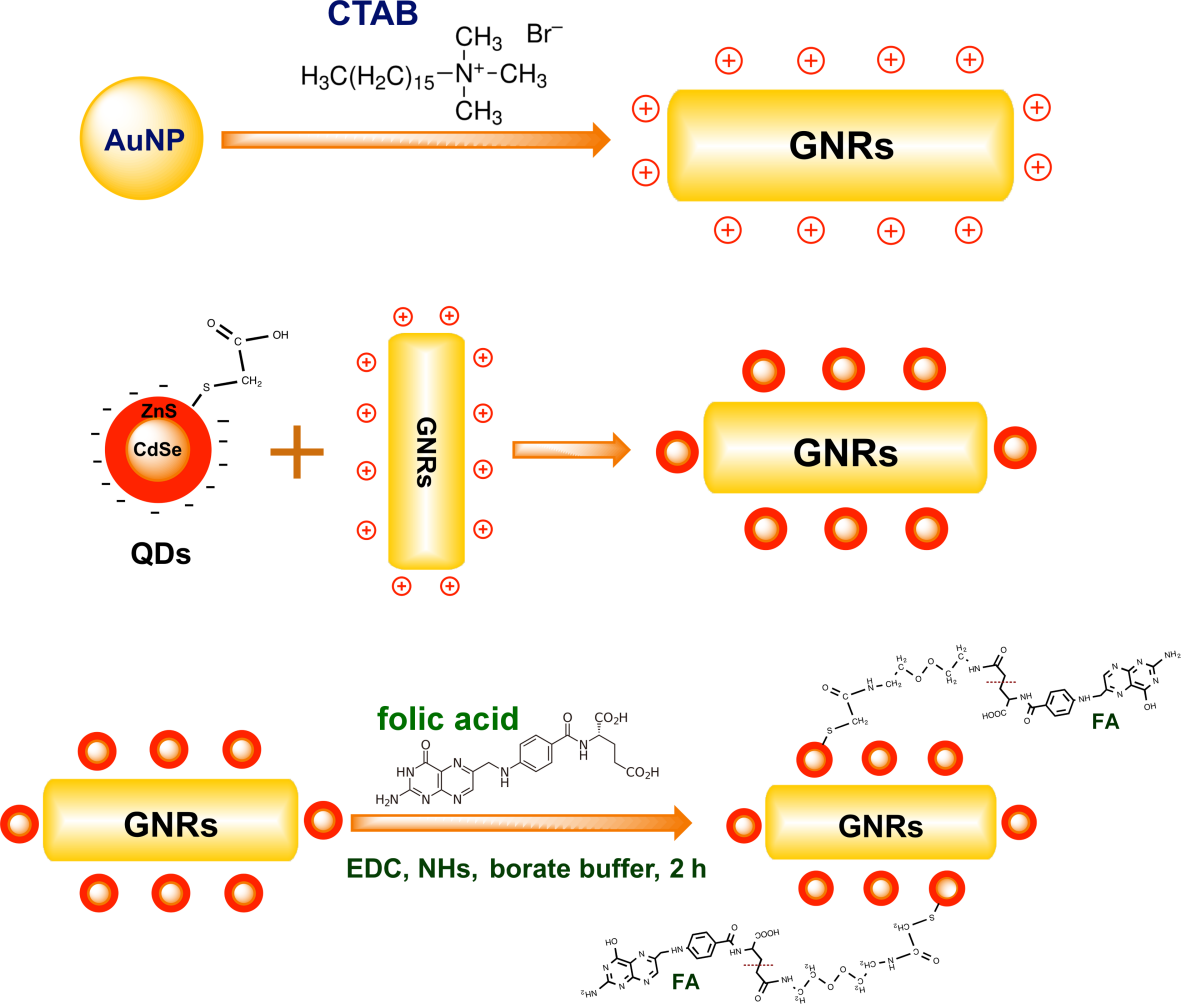
Schematic of GNR@CdSe/ZnS and GNR@CdSe/ZnS@FA.

The scheme for the synthesis of GNR@CdSe/ZnS and GNR@CdSe/ZnS@FA is illustrated in Figure 1. Firstly, the CdSe/ZnS QDs bind with GNRs kept at a 5 nm distance using the combined strong electrostatic adsorption. Secondly, FA was conjugated with this composite nanoparticle for biological applications, where the FA renders the nanoparticle useful for the specific targeting of cancer cells [28–29].

According to current knowledge, when bulk semiconductor nanomaterials are irradiated with light of a certain wavelength, the valence electrons transition back to the conduction band. Most of the conduction band electrons fall back into the deep electron traps and nonradiative transitions take the form of quenching. Semiconductor QDs with relatively shallow surface electron traps and holes can easily capture electrons to the conduction band. At the same time, the transition radiation returns to the valence band, emitting a photon. The PL characteristics are, therefore, better than in materials which have deep traps in the bulk semiconductor material. This finding suggests that when metal or chemical materials are doped with QDs, the number of surface trap states (which can lead to exponential PL decay) is affected by the surface passivation and by the degree of quantum confinement. In CdSe/ZnS QDs with higher defect densities, binding with GNRs having very heterogeneous energetic metal-derived states showed that their PL could be characterized by band edge dispersion in different sizes of CdSe/ZnS QDs and GNRs and that the PL contribution varied in these new states. The physical or chemical origin of these features was not clear, and this will be a subject of future research. We chose the longer absorption peak of the GNRs as equivalent to the QD PL emission peak. When the QDs and GNRs are coupled together, two kinds of nanostructured particles were combined by mutual electrostatic adsorption. The PL of the QDs could then be used as the light source to excite the LSPR of the GNRs in a way that the QD PL is enhanced when the GNR LSPR interacts with the QD PL plane wave.

**Figure 2:**
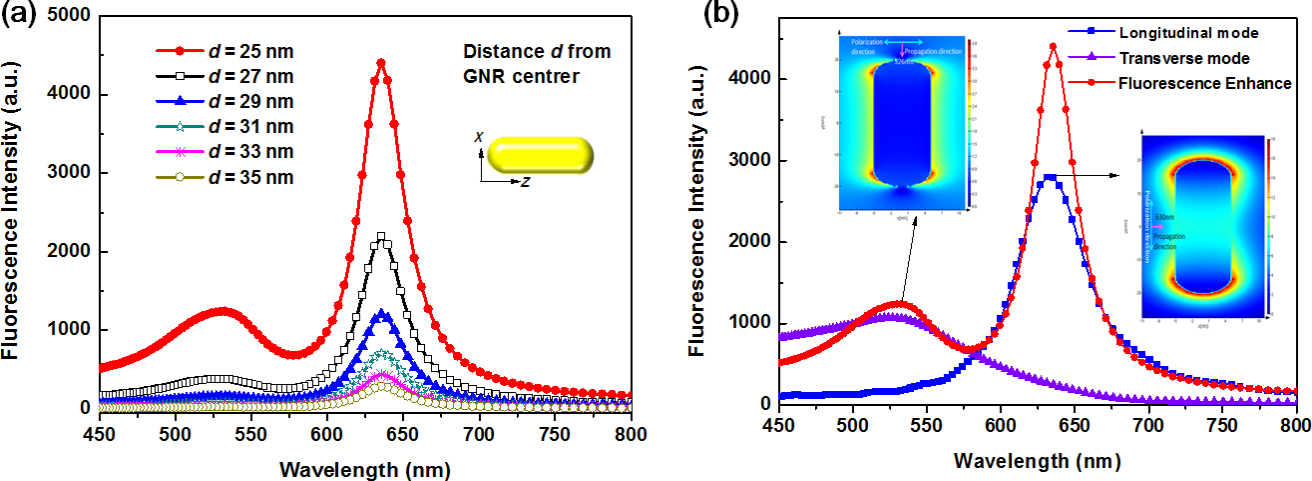
The simulation results of (a) photoluminescence enhancement as a function of wavelength and (b) wavelength and photoluminescence of GNRs under polarized light. The inset images inserted are the FDTD simulation of the electric field intensity distribution (indicated by the color bar) of the gold nanorods.

As shown in Figure 2a, from the FDTD simulation results we can see that (1) the highest PL enhancement, at 630 nm, occurred when *d* (*d* is the dipole-source center to GNR-center length) was set at 25 nm and (2) there is a slight enhancement at 526 nm, which is the short axis of the GNRs. That means when the QD emission is overlaid with the longer absorption peak of the GNRs, the PL enhancement will be maximized. 5 nm was found to be the optimal distance between GNRs and QDs.

To more clearly determine that the dipole source is the actual source of the PL enhancement, we use light polarization directed along the long and short axes of the GNRs to stimulate the longitudinal and transverse modes, respectively. As shown in Figure 2b, which shows the FDTD simulation results, we can see that the peak at 630 nm (corresponding to PL enhancement) comes from GNR longitudinal excitation. The peak at 526 nm originates from the transverse mode GNR excitation. This enhanced photoluminescence is due to the PL emission and LSPR of the QDs and as a far-field dipole.

**Figure 3:**
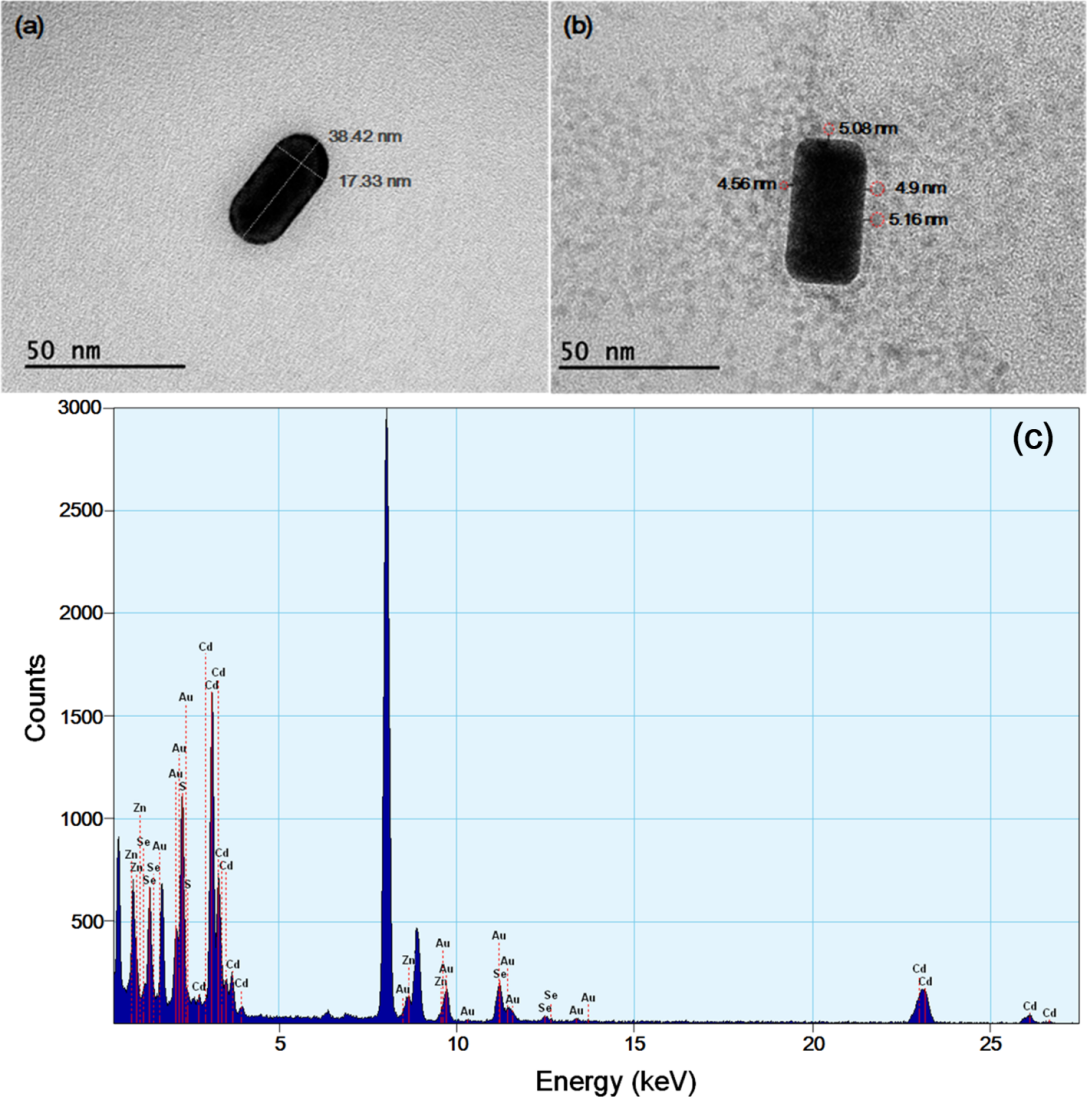
(a) TEM image of a GNR; (b) TEM image of GNR@CdSe/ZnS; (c) energy dispersive X-ray spectroscopy pattern of GNR@CdSe/ZnS.

The size and morphology of the GNRs and GNR@CdSe/ZnS was characterized using transmission electron microscopy (TEM), and the results are shown in Figure 3a and 3b. The average diameter of the CdSe/ZnS QDs is 8 ± 1 nm, where the corresponding TEM image is shown in Supporting Information File 1, Figure S2. The aspect ratio (length/diameter) is approximately 2.2, where the short and long axes length of the nanorods is around 17 nm and 38 nm, respectively, and the average distance between GNRs and QDs was around 5 nm, as shown in Figure 3b. The energy dispersive X-ray spectroscopy (EDS) pattern of GNR@CdSe/ZnS is given in Figure 3c. The result was consistent with the atomic ratio of the structure of GNR@CdSe/ZnS. The presence of Cd, Au, Zn, and Se is clear, and in our work, CdCl_2_, Se powder, ZnCl_2_, and Na_2_S were used as Cd, Se, Zn, and S sources, respectively. On the other hand, the Au peak comes from the GNRs. The results agree well with the synthesis chemical ratio, as explained in the Experimental section, suggesting that GNR@CdSe/ZnS nanoparticles were successfully synthesized by the described synthetic route.

**Figure 4:**
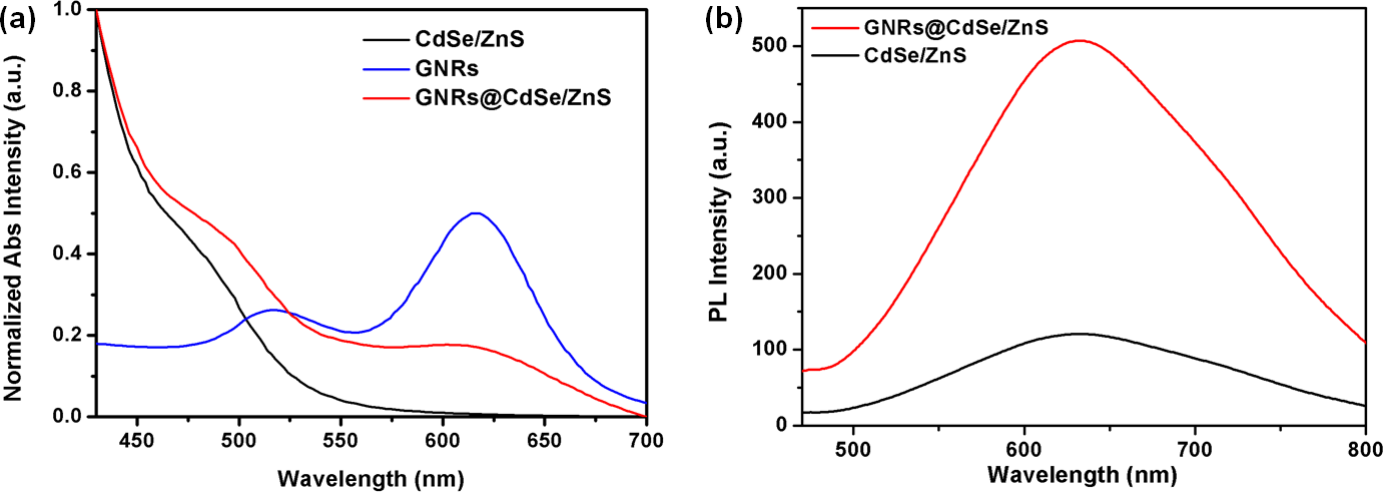
(a) Absorption spectrum of CdSe/ZnS, GNRs, and GNR@CdSe/ZnS; (b) PL spectrum of CdSe/ZnS and GNR@CdSe/ZnS.

The absorption and PL spectrum of CdSe/ZnS QDs and GNRs and the PL spectra of CdSe/ZnS QDs and GNR@CdSe/ZnS were acquired at room temperature as they were prepared, and the spectrum of water was measured as a reference. Figure 4a shows the absorption spectrum of CdSe/ZnS GNRs and GNR@CdSe/ZnS, where it can be seen that there are two peaks in the GNR absorption spectrum: the transverse surface plasmon resonance (TSPR) peak located at 510 nm and the LSPR peak located at 628 nm. Figure 4b shows the PL spectrum of CdSe/ZnS QDs where the peak emission is around 630 nm and the GNR absorption overlaps with the emission of the CdSe/ZnS QDs. The PL emission intensity of GNR@CdSe/ZnS was more than four times that of the CdSe/ZnS QDs, which implies that after binding with GNRs the PL intensity of CdSe/ZnS QDs was enhanced.

**Figure 5:**
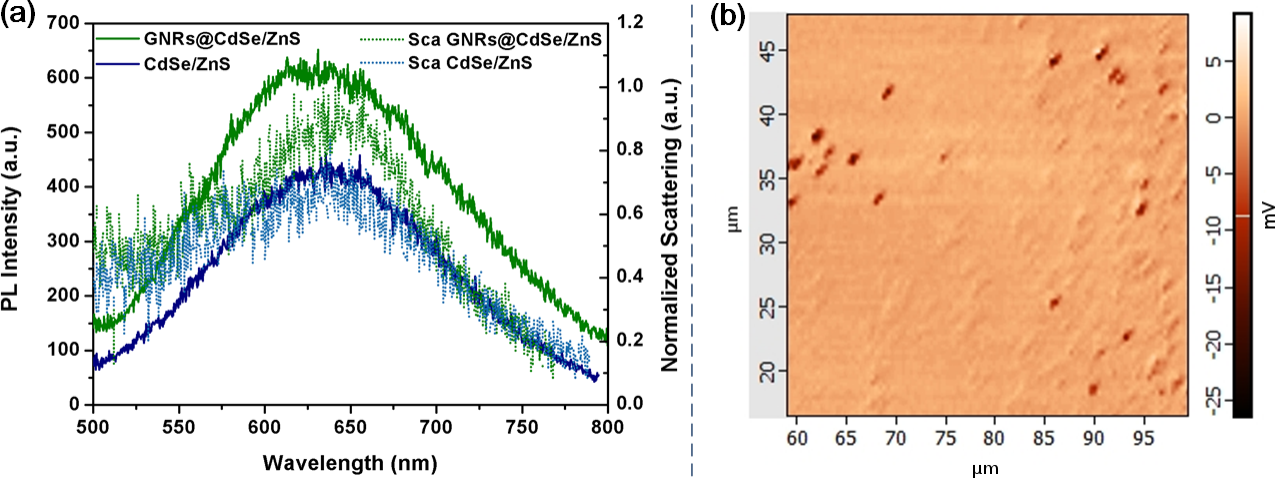
(a) PL and scattering spectrum from single CdSe/ZnS and GNR@CdSe/ZnS nanoparticles; (b) optical confocal scanning images of GNR@ CdSe/ZnS.

We performed additional optical characterization of a single GNR@CdSe/ZnS nanoparticle at room temperature. We diluted the GNR@CdSe/ZnS solution then placed it on glass to obtain a single particle per 1 × 1 μm area. The experimental microspectroscopy equipment was used to collect the integrated, white light, dark-field scattering, as well as the PL spectra and the atomic force microscopy techniques. We measured the scattering and the PL emission signal from a single particle. The measurements were taken under continuous wave laser excitation at a wavelength of 532 nm with a laser power of about 100 μW. The confocal scanning PL image of GNR@CdSe/ZnS is plotted in Figure 5, which was acquired from a sample region of 40 × 40 μm, where each dark spot corresponds to a single GNR@CdSe/ZnS nanoparticle.

Figure 5a shows the experimental PL and scattering spectrum of single particles of CdSe/ZnS QDs and GNR@CdSe/ZnS. Figure 5b shows optical confocal scanning images of GNR@CdSe/ZnS. When the GNRs were added to the QD solution, the PL emission intensity of GNR@CdSe/ZnS increased two times as compared with CdSe/ZnS. Additionally, compared to the PL spectrum in Figure 4b, the PL enhancement of a single particle is lower than that of the sample in the ensemble solution. Although still bright, this test also confirmed that a single GNR@CdSe/ZnS nanoparticle could serve as a single photon source.

**Figure 6:**
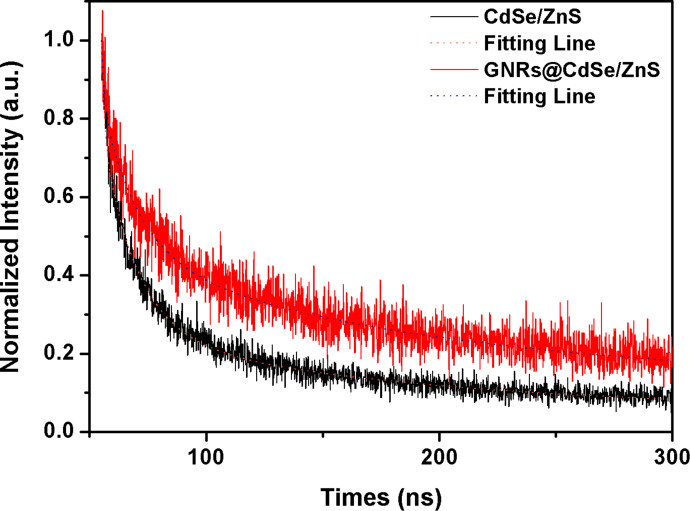
Photoluminescence lifetime spectrum of CdSe/ZnS and GNR@CdSe/ZnS.

PL lifetime is also an important parameter in studying the photoelectric properties and surface chemistry of nanoparticles. A multiexponential decay curve provides evidence of the presence of nonradioactive or radiative recombination. The lifetime of GNR@CdSe/ZnS and CdSe/ZnS was obtained by using a fluorescence lifetime spectrometer (FLS980) with a detection wavelength range of 200 to 1700 nm and an excitation wavelength of 450 nm. The spectra are shown in Figure 6.

These lifetime measurements confirmed that the lifetime of GNR@CdSe/ZnS was longer than that of CdSe/ZnS, as shown by the data in Table 1. Here, *B* is a percentage of the lifetime and *t* is the lifetime. From Figure 4 we can see that the band edge of the QDs is near 500 nm and the band edge of our CdSe/ZnS sample is calculated to be about 2.48 eV, which is larger than the value of 1.76 eV for CdSe, thus the PL of CdSe/ZnS is surface defect emission. This greater lifetime resulted from the trapping states caused by surface defects located within the bandgap, which led to a rise of nonradioactive recombination. When the GNRs were doped with CdSe/ZnS QDs, more radiative and nonradioactive processes occurred at the particle surface, which contributed to an enhanced lifetime.

**Table 1:** Lifetime data of the samples.

Sample	*B*_1_	*t*_1_ (ns)	*B*_2_	*t*_2_ (ns)	*B*_3_	*t*_3_ (ns)	Average lifetime (ns)

CdSe/ZnS	0.0702	1.1411	0.2403	14.885	0.6896	128.5203	92.2753
GNR@CdSe/ZnS	0.0342	1.1047	0.1303	15.5347	0.8354	134.5797	114.5012

**Figure 7:**
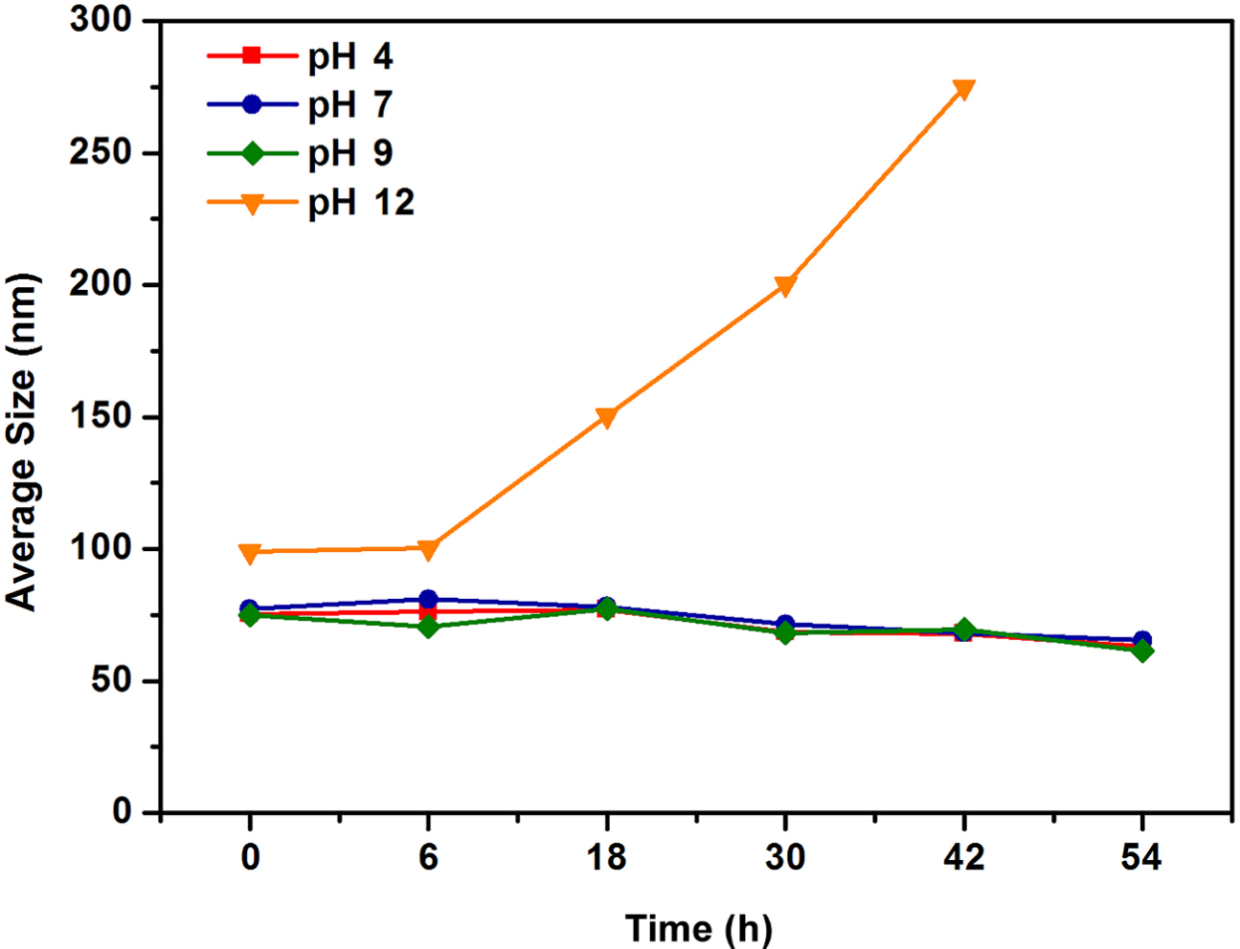
The colloidal stability of GNR@CdSe/ZnS@FA.

To make this multifunctional nanomaterial more biocompatible and specific to the cancer cell, we conjugated the FA with GNR@CdSe/ZnS nanoparticles. Firstly, we performed a colloidal stability study in which their hydrodynamic sizes were monitored using the dynamic light scattering technique at room temperature. The results shown in Figure 7 indicated relatively stable conditions of GNR@CdSe/ZnS@FA formulations when the pH values were 4, 7, and 9, and that the samples were relatively stable in the alkaline environment. The size of GNR@CdSe/ZnS@FA did not show significant change during the measurement, but when the pH value changed to 12, the size of GNR@CdSe/ZnS@FA increased. The size increase was caused by aggregation, which was due to the high pH solution being rich in hydroxide ions (OH^−^), which facilitates the carboxyl groups of the surface of nanoparticles. However, still below 300 nm, this size is also suitable for bioimaging, and the results suggest that the GNR@CdSe/ZnS@FA sample could be applied in biological applications.

**Figure 8:**
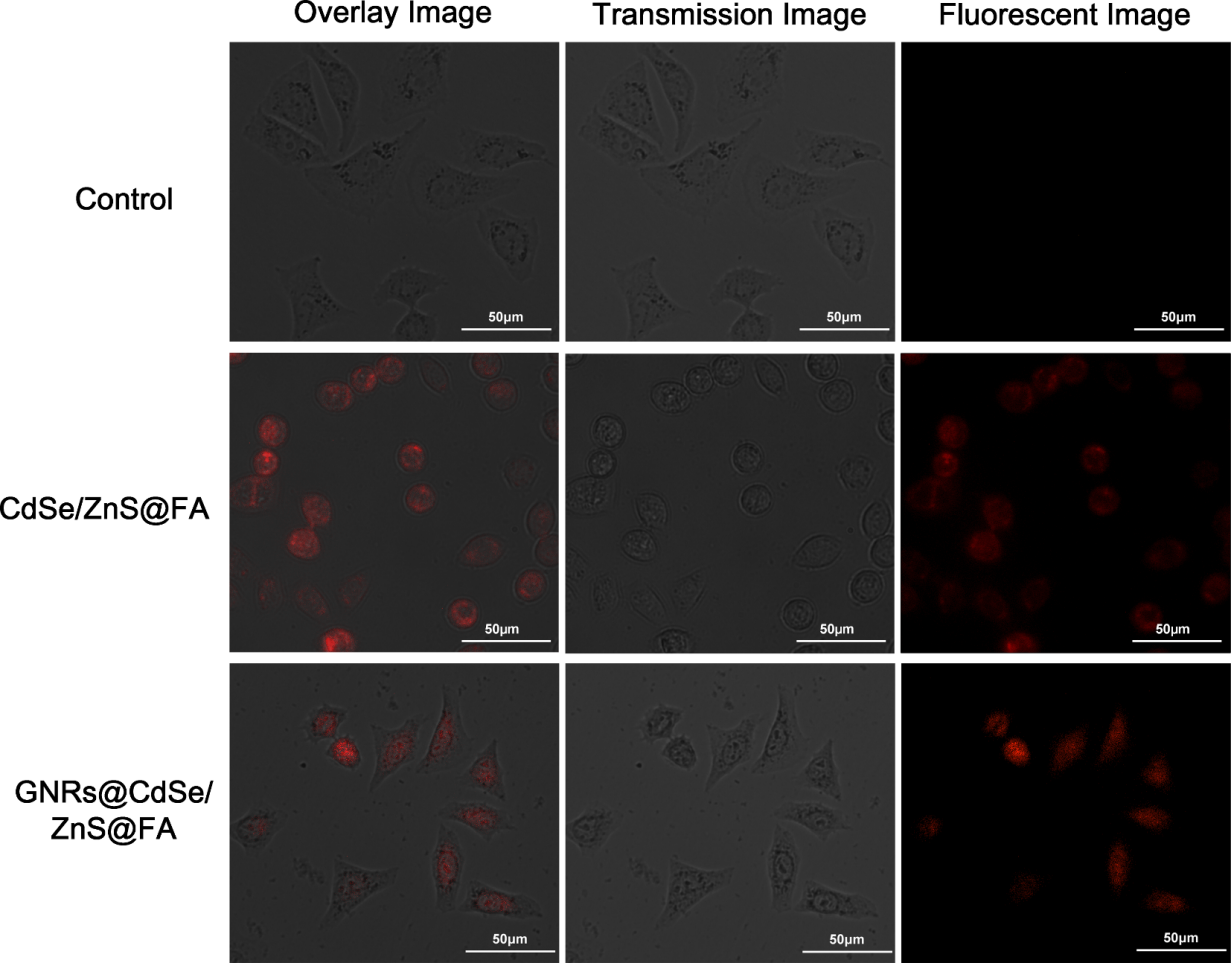
Microscopy images of MCF-7 breast cancer cell labelled with CdSe/ZnS@FA and GNR@CdSe/ZnS@FA.

Over the past few years, the synthesis of CdSe and CdSe/ZnS QDs for in vitro imaging has received significant attention. To confirm that GNR@CdSe/ZnS can be used in cell imaging studies, after conjugation with FA, we performed a MCF-7 breast cancer cell targeting imaging study, and the results as shown in Figure 8. Robust cellular uptake could be obtained from the CdSe/ZnS@FA and GNR@CdSe/ZnS@FA treated samples. The obvious luminescence and staining appearing in Figure 8 was due to the accumulation of functionalized nanoparticles in the cells, and there was no sign of any damage to the cell, demonstrating passive uptake in MCF-7 breast cancer cells using CdSe/ZnS@FA and GNR@CdSe/ZnS@FA. However, because the PL intensity and cell morphology of GNR@CdSe/ZnS@FA is better than CdSe/ZnS@FA, the in vitro imaging results confirm that these nanoparticles are suitable to be used for near-infrared imaging and cancer therapy.

## Conclusion

We have developed a multifunctional nanomaterial GNR@CdSe/ZnS and enhanced the PL intensity using the surface plasmon resonance of GNRs. The experimental results correlated well with the theoretical simulation. The results of the biological detection study indicated that this nanomaterial is biocompatible and that there is significant PL signal for cell imaging. This research is promising for future nanophotonics and biophotonics applications.

## Experimental

### Materials and instrumentation

Hexadecyltrimethylammonium bromide (CTAB, >98.0%), L-ascorbic acid (BioUltra, ≥99.5%), silver nitrate (AgNO_3_, >99%), gold(III) chloride trihydrate (HAuCl_4_·3H_2_O, 99%), 3-mercaptopropionic acid (MPA, ≥99%), *N*-ethyl-*N'*-(3-dimethylaminopropyl)carbodiimide (EDC, 99%), and *N*-hydroxysuccinimide (NHs, 99%), cadmium chlorideanhydrous (CdCl_2_, 99.999%), selenium powder (Se, 99.8%), sodium borohydride (NaBH_4_, 99.99%), and zinc chloride anhydrous were purchased from Sigma-Aldrich. Sodium sulphide (Na_2_S, 60–63%) was purchased from Acros Organics, and anhydrous ethanol, sodium hydroxide (NaOH, AR) and hydrochloric acid (HCl, AR) were purchased from Sinopharm Chemical Reagent Co., Ltd. All chemicals were used as received without further purification. Deionized (DI) water used in all the studies was purified by a Milli-Q water purification system.

### Characterization

The PL emission spectrum of the QDs was measured with a Cary Eclipse fluorescence spectrophotometer (Agilent, CA, USA) at an excitation wavelength of 430 nm. UV–visible absorption spectra were collected using a Cary 5000 spectrophotometer (Agilent, CA, USA). The lifetime of the nanoparticles was measured with an FLS980 spectrometer (Edinburgh instruments, UK). The morphology and size of the GNRs and QDs were obtained with an FEI Tecnai G2 F20 S-TWIN TEM operating at an accelerating voltage of 200 kV, and the samples were loaded into a quartz cell for the measurements. The TEM specimens were prepared by drop casting the sample dispersion onto an amorphous carbon-coated 300 mesh copper grid.

### Synthesis of GNRs

To synthesize the GNRs, the seed-mediated growth method in CTAB solution was applied, as previously discussed [30–31]. The seed solution was prepared using 5 mL of a 0.2 M CTAB solution and 5 mL of 0.1 mM HAuCl_4_. Following this, 0.6 mL of ice-cold 0.01 M NaBH_4_ solution was quickly added to the HAuCl_4_^−^ CTAB solution and vigorously stirred for 3–5 min. This caused the solution color to change from yellow to light brown. The solution was stored at 37 °C for 30 min before use. The growth solution was prepared by mixing 800 μL of 25 mM HAuCl_4_ and 20 mL of HPLC water. Following this, 400 μL of 4.0 mM AgNO_3_ solution was added to the HAuCl_4_ solution, and then 20 mL of 0.2 M CTAB and 800 μL of 0.08 M ascorbic acid were added. This solution was kept in a water bath at 37 °C, and the solution became clear and colorless after gentle stirring. 96 μL of the seed solution was added to the growth solution, and it was then left undisturbed at 37 °C for 24 h. The final solution was centrifuged at 8000 rpm for 10 min twice then redispersed in 0.02 M CTAB solution for later use.

### Synthesis of CdSe/ZnS QDs

A selenium precursor was prepared by reducing 19.4 mg of selenium powder with 40 mg of sodium borohydride (NaBH_4_) in 1 mL of nitrogen-saturated DI water at room temperature. The mixture was stirred for 1–2 h until it became colorless.

MPA-CdSe QDs were synthesized by the previously reported method. Briefly, 366 mg of CdCl_2_, 440 μL of MPA, and 50 mL of nitrogen-saturated water were loaded into a three-necked flask under stirring. The pH was adjusted to 10 by dropwise addition of sodium hydroxide solution. The Se precursor was then injected into the mixture under nitrogen atmosphere, and the reaction mixture was slowly heated under nitrogen atmosphere to 98 °C. After 2 h, the mixture solution was collected as MPA-CdSe QDs for ZnS shell-coating synthesis. The ZnCl_2_ was prepared by loading 68 mg of ZnCl_2,_ 100 μL of MPA, and 30 mL of nitrogen-saturated water into a three-necked flask under stirring. The pH was adjusted to 10 by adding 1 M of sodium hydroxide solution. The ZnCl_2_ solution was then added to the CdSe solution at 60 °C, and after the mixture was heated to 90 °C, 60 mg (2 mL) of Na_2_S was added to the ZnCl_2_-CdSe solution and maintained at 98 °C for 1 h. Finally, the solution was cooled to room temperature and separated by the addition of ethanol and two cycles of centrifugation.

### Synthesis of GNR@CdSe/ZnS nanoparticles

The GNR solution (5 mL, 5 OD/mL) was centrifuged at 8000 rpm for 10 min and the supernatant was discarded. The GNR precipitate was redispersed in HPLC water to obtain 0.1 OD/mL. Subsequently, 1 mL of the CdSe/ZnS (1 OD/mL) mixture was added to 10 mL of the GNR solution, and the mixture was left under stirring overnight to obtain the GNR@CdSe/ZnS composite nanoparticles.

### Synthesis of GNR@CdSe/ZnS@FA nanoparticles

From the stock solution, 2 mL of 4 mg/mL aqueous GNR@CdSe/ZnS dispersion was mixed with 400 µL of 10 mM EDC solution and incubated for 2 min. 400 µL of 10 mM NHs was then added to the mixture and stirred for 10 min. Next, 1.2 mL of folic acid in DMSO solution (2 mg/mL) was added to this mixture and stirred at room temperature for 2 h to allow the folic acid to covalently couple with the nanoparticles. After two hours of stirring, the bioconjugated nanoparticles were purified (removing excess by-products) via centrifugation. The QD precipitate was redispersed in HPLC water for the bioimaging studies.

### Simulation study of photoluminescence enhancement

The simulation study was based on Parseval’s equation, where the electromagnetic fields can be expressed as described in Equation 1 and Equation 2, and the dipole integral is according to the perpendicular [26].

[1]
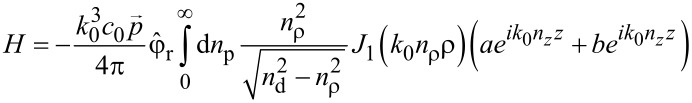


[2]
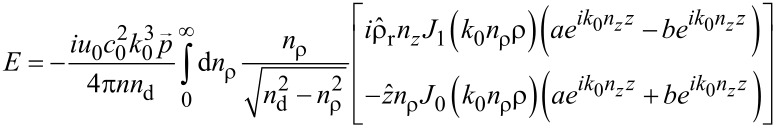


The normalized total output power of photoluminescence of can be expressed as [26]

[3]
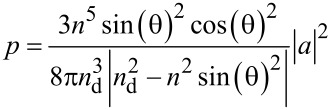


[4]
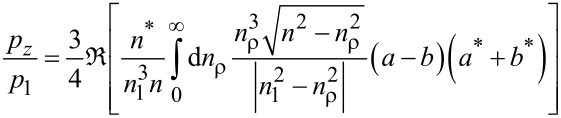


[5]
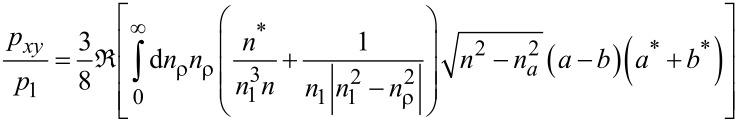


We also simulated the PL enhancement of GNR@CdSe/ZnS using FDTD software, and we used these simulation results to further study the PL enhancement caused by LSPR coupling effect. The FDTD simulation results show that the quantum mechanical decay rate of the inhomogeneous environment is related to the classical power radiated by the dipole in the same environment, which only occurs in the radiation of an atomic dipole transition. Specifically, we have the following relationship:

[6]
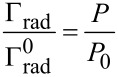


where Γ_rad_ is the decay rate, *P* is the radiated power in an inhomogeneous environment (with the nanoparticle near the dipole source), 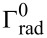 is the decay rate, and *P*_0_ is the power radiated in a homogeneous environment (only the dipole source). When the isolated emitter is coupled to the nanoantenna, its quantum efficiency is modified as,

[7]
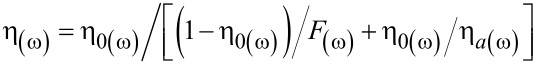


In this relation,


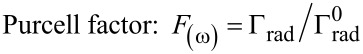



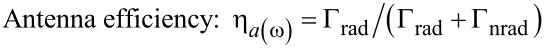


The PL enhancement, *S*/*S*_0_, is then

[8]
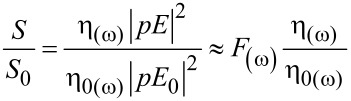


where *p* is the transition electric dipole moment. From the above equations, we can see the PL enhancement effected by the *F*_(ω)_ and η*_a_*_(ω)_. Here η_0(ω)_ is the quantum efficiency related to the enhancement as a reference [22–23].

For FDTD simulations we use a dipole source to simulate the QDs, which emit in the wavelength range from 450 nm to 800 nm with polarization along the long axis direction *z*. *d* is the distance between the dipole source center and the GNR center, and the length of the GNRs was fixed at 40 nm.

### In vitro cell imaging study

MCF-7 breast cancer cells (American Type Culture Collection) were cultured with Dulbecco’s Modified Eagle’s Medium (DMEM, Hyclone), supplemented with 10% fetal bovine serum (FBS, Hyclone), penicillin (100 µg mL^−1^, Gibco), and streptomycin (100 µg mL^−1^, Gibco) in a humidified environment (37 °C, 5% CO_2_). Before treating with nanoparticles, the cells were seeded onto cover glass in a six-well plate with DMEM. The prepared CdSe/ZnS@FA QDs and GNR@CdSe/ZnS@FA nanoparticles were then diluted with PBS buffer (pH 7.2) solution to a concentration of 500 μg/mL. Next, the cells were treated with the CdSe/ZnS@FA QDs and GNR@CdSe/ZnS@FA nanoparticles for 4 h. After 4 h of incubation, the treated cells were washed with PBS buffer three times. A Leica DMI 3000 inverted microscope with a 10× lens was used for the cell imaging study, and the excitation and emission wavelengths were 532 nm and 630 nm, respectively.

## Supporting Information

File 1 Relative cell viability of MCF-7 breast cancer cells treated with different concentrations (6.25–100 μg/mL) of GNR@CdSe/ZnS for 24 h. TEM image of CdSe/ZnS QDs.
