# Supplementary material for: Surface plasmon resonance enhancement of photoluminescence intensity and bioimaging application of gold nanorod@CdSe/ZnS quantum dots
Source: Beilstein J Nanotechnol. 2019 Jan 3;10:22–31. doi: 10.3762/bjnano.10.3 (PMC6334792; doi:10.3762/bjnano.10.3)
Supplement: File 1 — Relative cell viability of MCF-7 breast cancer cells treated with different concentrations (6.25–100 μg/mL) of GNR@CdSe/ZnS for 24 h. TEM image of CdSe/ZnS QDs. [file Beilstein_J_Nanotechnol-10-22-s001.pdf]

BEILSTEIN JOURNAL OF NANOTECHNOLOGY

Supporting Information

for

Surface plasmon resonance enhancement of photoluminescence intensity and bioimaging application of gold nanorod@CdSe/ZnS quantum dots

Siyi Hu, Yu Ren, Yue Wang, Jinhua Li, Junle Qu, Liwei Liu, Hanbin Ma and Yuguo Tang

Beilstein J. Nanotechnol. **2019,** *10,* 22–31. doi:10.3762/bjnano.10.3

Relative cell viability of MCF-7 breast cancer cells treated with different concentrations (6.25–100 µg/mL) of GNR@CdSe/ZnS for 24 h. TEM image of CdSe/ZnS QDs

License and Terms: This is a supporting information file under the terms of the Creative Commons Attribution License (http://creativecommons.org/licenses/by/4.0). Please note that the reuse, redistribution and reproduction in particular requires that the authors and source are credited.
The license is subject to the *Beilstein Journal of Nanotechnology* terms and conditions: (https://www.beilstein-journals.org/bjnano)

Cell viability studies were performed by using the MTT assay method. Briefly, the cells we applied are MCF-7 breast cancer cells. Firstly, we seeded cells into a 96-well plate with cell density of 50% for 24 h at 37 °C with 5% CO_2. Secondly, we added different concentrations of GNRs@CdSe/ZnS nanoparticles (6.25–100 μg/mL) in the cells, then incubated for another 24 h at 37 °C with 5% CO_2. Thirdly, 18 μL of MTT (5 mg/mL) solution was added to each well, then the cells were incubated for another 4 h at 37 °C with 5% CO_2. Fourthly, 150 μL of dimethyl sulfoxide (DMSO, Sigma) was added into the each wells. The absorbance of the samples at 490 nm were measured using microplate reader (Infinite M200 PRO, Tecan). The cell viability was obtained by normalizing the absorbance of the sample wells against that of the control wells.

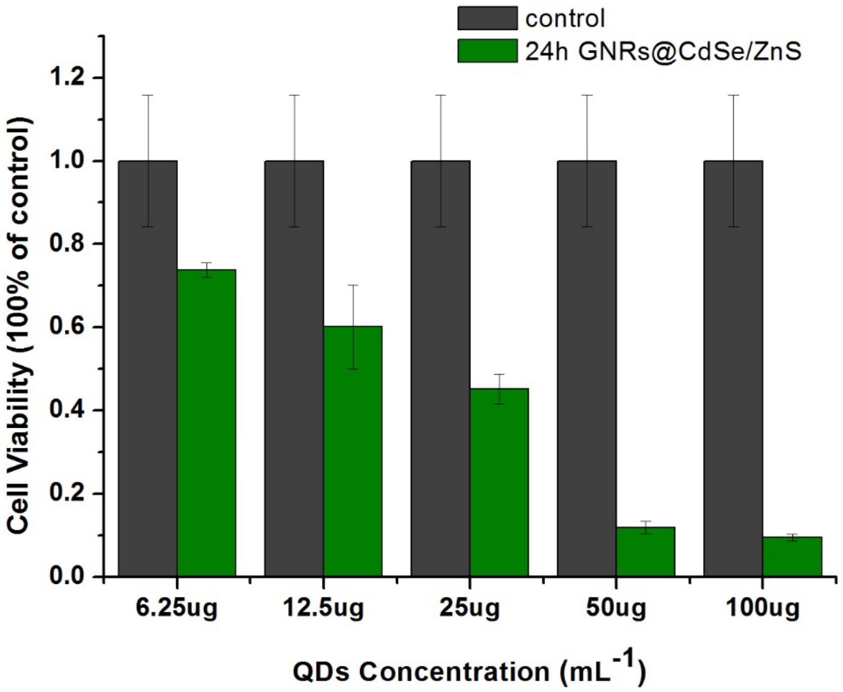

Figure S1: Relative cell viability of MCF-7 breast cancer cell treated with different concentrations (6.25–100 μg/mL) of GNRs@CdSe/ZnS for 24 h.

The average size of the CdSe/ZnS QDs is 8 ± 1 nm, the TEM image of QDs as shown in below.

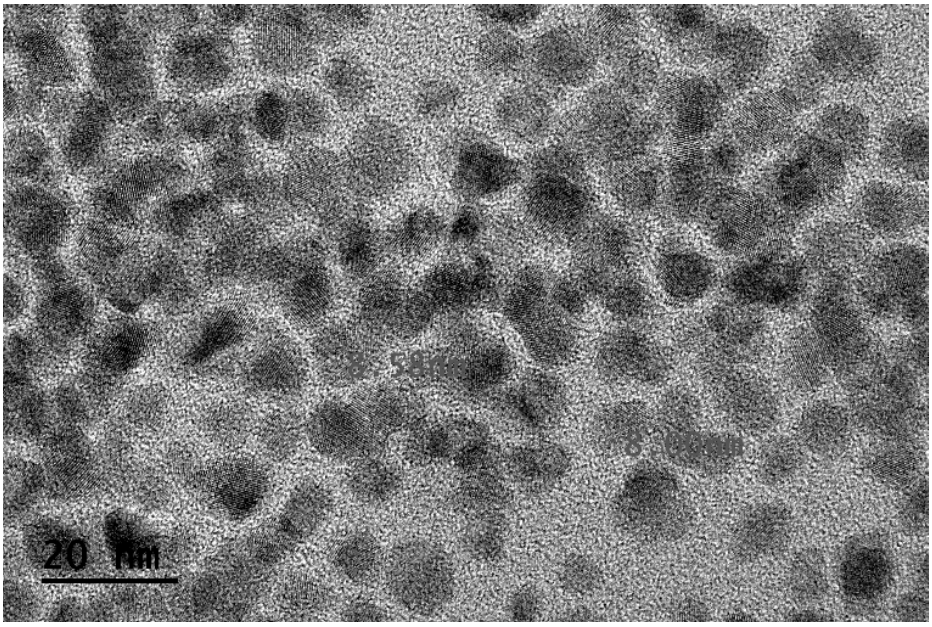

Figure S2: TEM image of CdSe/ZnS QDs.
